# Lifestyle Adjustments in Long-COVID Management: Potential Benefits of Plant-Based Diets

**DOI:** 10.1007/s13668-021-00369-x

**Published:** 2021-09-10

**Authors:** Maximilian Andreas Storz

**Affiliations:** Centre for Complementary Medicine, Institute for Infection Prevention and Hospital Epidemiology, Faculty of Medicine, University of Freiburg, Freiburg im Breisgau, Germany

**Keywords:** SARS-CoV-2; Long-COVID, Plant-based diet, Vegan, Nutrition, Economic burden

## Abstract

***Purpose of Review*:**

The SARS-CoV-2-pandemic has caused mortality and morbidity at an unprecedented global scale. Many patients infected with SARS-CoV-2 continue to experience symptoms after the acute phase of infection and report fatigue, sleep difficulties, anxiety, and depression as well as arthralgia and muscle weakness. Summarized under the umbrella term “long-COVID,” these symptoms may last weeks to months and impose a substantial burden on affected individuals. Dietary approaches to tackle these complications have received comparably little attention. Although plant-based diets in particular were shown to exert benefits on underlying conditions linked to poor COVID-19 outcomes, their role with regard to COVID-19 sequelae is yet largely unknown. Thus, this review sought to investigate whether a plant-based diet could reduce the burden of long-COVID.

***Recent Findings*:**

The number of clinical trials investigating the role of plant-based nutrition in COVID-19 prevention and management is currently limited. Yet, there is evidence from pre-pandemic observational and clinical studies that a plant-based diet may be of general benefit with regard to several clinical conditions that can also be found in individuals with COVID-19. These include anxiety, depression, sleep disorders, and musculoskeletal pain. Adoption of a plant-based diet leads to a reduced intake in pro-inflammatory mediators and could be one accessible strategy to tackle long-COVID associated prolonged systemic inflammation.

***Summary*:**

Plant-based diets may be of general benefit with regard to some of the most commonly found COVID-19 sequelae. Additional trials investigating which plant-based eating patterns confer the greatest benefit in the battle against long-COVID are urgently warranted.

## Introduction

The SARS-CoV-2-pandemic has caused mortality and morbidity at an unprecedented global scale [1]. On May 30, 2021, the World Health Organization reported over 169 million cumulative cases, with over 3.5 million deaths worldwide [2]. While early attention focused on acute illness management, recent studies suggested that some patients continue to experience symptoms and complications after the acute phase of infection [3, 4]. These symptoms may last weeks to months after initial recovery [5] and were summarized under the umbrella term “long-COVID” [6].

Up to 80% of patients infected with SARS-CoV-2 develop at least one long-term symptom [5], including fatigue, headaches, cognitive disturbances, sleep difficulties, anxiety, and depression as well as arthralgia, muscle weakness, dyspnea, and chest pain [1, 5, 7–10].

Affected individuals may require long-term support as studies suggest that many COVID-19 survivors will face impairments regarding their mental health or physical function far beyond hospital discharge [11]. Therefore, an increasing number of physicians have called for coordinated attempts to understand the overall survivorship burden associated with this condition [12] leading to a debate about how healthcare professionals should manage long-COVID [13].

The care of this particularly vulnerable patient population requires a multidisciplinary approach [14], and some experts suggest that resource allocation should prioritize rehabilitation and psychological support over advanced diagnostics and specialist respiratory service [13]. Cost-efficient and widely applicable public health strategies concomitantly improving many of the aforementioned long-COVID related symptoms are urgently warranted.

Although the World Health Organization emphasizes that alterations in diet have strong effects on health throughout life [15], dietary approaches to tackle long-COVID-related symptoms have received comparably little attention [16, 17].

Some authors and non-profit organizations, including the Physicians Committee for Responsible Medicine, highlighted the benefits of a healthy plant-based diet in fighting underlying conditions linked to poor COVID-19 outcomes [18, 19]. As defined by Ostfeld, a plant-based diet consists of (minimally processed) fruits, vegetables, whole grains, and legumes, while excluding all animal products (such as red meat, poultry, fish, and dairy) [20]. While there are varying definitions of plant-based eating patterns [21], all of them are defined in terms of low frequency of consumption of animal foods [22]. Instead, plant-based diets emphasize vegetables, legumes, fruits, whole grains, nuts, herbs, and seeds [23]. Such a diet is abundant in fiber, antioxidants, and phytochemicals, while free of cholesterol and low in (saturated) fat and pro-inflammatory animal-derived molecules [24–27].

Recent research centered around whole-food plant-based nutrition, which is characterized by an unrestricted consumption of (whole) plant-based foods and the exclusion of both animal-based and processed foods, such as added oils, fried products, and sugary packaged items [21]. In this review, the authors examine published work on health-benefits associated with such a plant-based dietary pattern and use the term “plant-based” to collectively refer to all plant-based eating patterns where the majority of energy is derived from plant foods. Where appropriate, specific terms (e.g., lacto-ovo-vegetarian or vegan) are used.

This narrative review considers nutrition as an accessible lifestyle modification that could reduce the burden of long-COVID on quality of life. This review also summarizes further studies that are necessary to support the postulated benefits of plant-based nutrition on long-COVID.

## Plant-Based Diets and COVID-19

Clinical studies examining the effects of a plant-based diet in COVID-19 prevention and management are (yet) scarce. A very recently published population-based case–control study in six countries investigated the association between dietary patterns and COVID-19. Following a plant-based dietary pattern was associated with 73% lower odds of moderate-to-severe COVID-19-like illness [28••]. In contrast, those individuals following “low carbohydrate, high protein diets” had a substantially greater odds of moderate-to-severe COVID-19. The reservation must be made, however, that conclusions of this study were based on a rather small sample of cases (in a selected cohort of healthcare workers at the COVID-19 frontline) and that diet was self-reported.

Whether a plant-based diet might be useful to alleviate symptoms related to long-COVID has not (yet) been examined in clinical trials. Nevertheless, there is evidence from epidemiological, observational, and clinical studies done in the pre-pandemic era that a plant-based dietary pattern may be of general benefit with regard to some clinical conditions that can also be found in individuals with COVID-19. These include fatigue, sleep disorders, headaches, anxiety, and depression as well as musculoskeletal pain.

## Mental Health

The COVID-19 pandemic has caused significant damage to public mental health [29]. Studies from China and Hong Kong revealed an increasing prevalence of depression and anxiety within the general public [30, 31]. In an early survey among Hong Kong citizens, 25.4% reported that their mental health had deteriorated since the pandemic [31]. Young US adults reported higher rates of loneliness, high levels of COVID-19-specific worry, and low distress tolerance [32]. In an Italian sample, 55% of participants presented a clinical score for at least one mental disorder [33].

Affected individuals could benefit from targeted nutritional interventions promoting plant-based eating patterns. Some studies suggest that emotional health is closely associated with fruit and vegetable consumption [34, 35]. While people with poor diets tend to be less happy and healthy [34], a dose–response relationship was found between daily servings of fruits and vegetables and both life satisfaction and optimism [36]. In particular, optimism, a much needed emotion in current times, was associated with higher vegetable consumption and greater serum carotenoid concentrations [37–39] (plant pigments naturally found in abundance in vegetables and fruits [40]).

Poor nutrition may contribute to poor mental health [41, 42], particularly with regard to depression and anxiety [43]. Individuals suffering from either condition reported lower fruit and vegetable intakes and consumed higher amounts of added sugars [44]. Processed foods including sweetened desserts, processed meats, refined grains, and high-fat dairy products were associated with increased odds of depression in the Whitehall II prospective cohort [45]. This was reinforced by a recent meta-analysis revealing an association between meat consumption and a moderately higher incidence of depression [46].

In contrast, vegetarians who regularly eat plenty of fruits and vegetables reported significantly fewer negative emotions compared to omnivores in a cross-sectional study in healthy Seven Day Adventists [47]. Vegetarians are also to less likely to suffer from depression [48]. The reservation must be made, however, that individuals on a plant-based diets may generally be healthier [47] and reported a higher quality of life in several studies [49, 50]. Thus, results from the aforementioned studies must be interpreted with great caution and do not allow to state unambiguously that fruit and vegetable intake directly influence mental health, as the reverse causation is also possible [35]. Yet, plant-based eating patterns are high in polyphenols and antioxidants, which were associated with beneficial effects on cognitive and mental health [51].

### Antioxidants and Polyphenols

Both are readily found in plant foods and were shown to influence cerebral blood flow, cellular energy metabolism, and modulate signaling pathways of molecules involved with brain plasticity [52–55]. Naturally occurring dietary polyphenols, found in apples, plums, cherries, onions, and tea, exhibit antidepressant activity with relatively low doses (0.3–2 mg/kg) and are an effective means to prevent (or delay) both anxiety and depression [53]. Quercetin, a polyphenol phytochemical compound found exclusively in plant foods [56, 57], is probably the most prominent example.

**Fig. 1:**
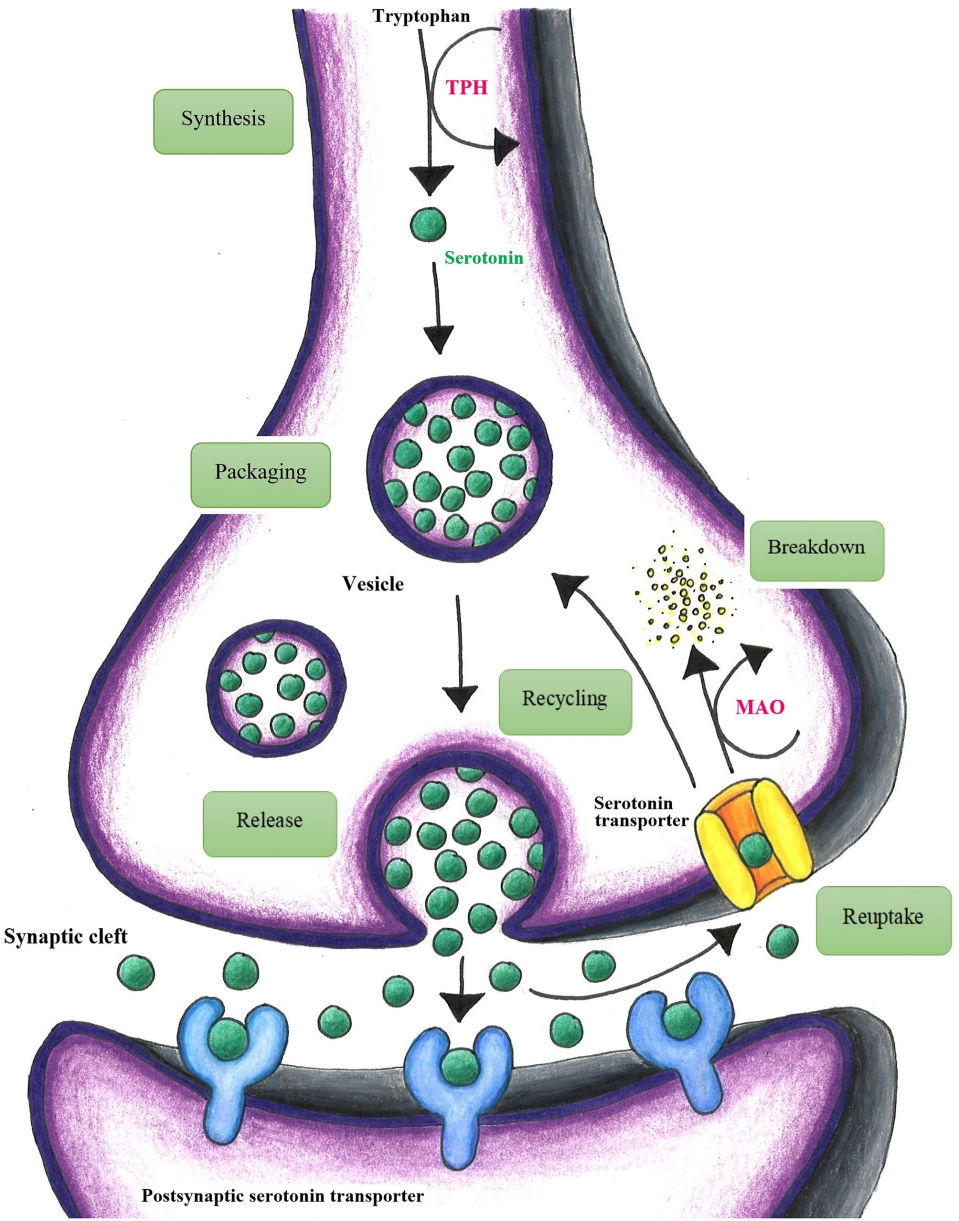
The serotonin theory of depression: a schematic illustration. Quercetin inhibits the key enzyme monoamine oxidase (MAO), thereby preventing the degradation of monoamine neurotransmitters such as serotonin, norepinephrine, and dopamine (modified from [59, 60])

Quercetin acts as a dietary monoamine oxidase (MAO) inhibitor, preventing the degradation of monoamine neurotransmitters such as serotonin, norepinephrine, and dopamine [58]. The serotonin theory of depression postulates that depression is the consequence of an excessive breakdown of monoamine neurotransmitters subsequent to a pathologically upregulated catalytic activity of MAO-A (Fig. 1) [59, 60].

Quercetin was shown to decrease this neurotransmitter breakdown [58] and prevented depression-like behavior in animal studies [61–63]. It is exclusively found in plant-foods; thus, a plant-based eating pattern abundant in fruits, vegetables, and legumes enhances its intake and has been proposed as a promising approach for the prevention and treatment of anxiety and depression [53]. Although quercetin also possesses antiviral properties [64, 65], it has not yet been tested in a clinical trial in patients suffering from COVID-19.

### Fatty Acids, Mental Health, and COVID-19

Another frequently encountered nutritional approach to improve mental health is the increased consumption or supplementation of omega-3 fatty acids [66]. Omega-3 fatty acids have anti-inflammatory and immunomodulatory properties [67, 68] and were tested in several recent trials in patients suffering from COVID-19. A higher omega-3 index was associated with a lower risk of death from COVID-19 [69], as well as with improvements in several parameters of respiratory and renal function in critically ill COVID-19 patients [70]. Moreover, omega-3 fatty acids have been suggested to strengthen psychological resilience during the COVID-19 pandemic [71].

Interventional studies investigating the role of omega-3 fatty acids in individuals with long-COVID are currently not existent. Although omega-3 fatty acids can be obtained from fatty fish [72], some smaller pre-pandemic studies suggested that a diet free of fish (and other animal products) may be better for mood improvement [73]. In 2012, Beezhold and colleagues randomized 39 omnivores to one of three groups: (1) a control group consuming meat, fish, and poultry daily, (2) a group consuming fish 3–4 times weekly but avoiding poultry and meat, and finally, (3) a lacto-ovo vegetarian group avoiding meat, fish, and poultry [74]. Within just 2 weeks, participants in the second group substantially increased their omega-3 intake (~ 270–364 mg/day of eicosapentaenoic acid (EPA) docosahexaenoic acid (DHA)). Their mood scores, however, remained unchanged (as measured by the Profile of Mood States questionnaire and the Depression Anxiety and Stress Scale). Although the vegetarian group reduced their EPA and DHA intake, their mood scores improved significantly. The authors concluded that consuming a diet high in fish may not beneficially affect mental state, an important consideration with regard to declining public mental health subsequent to the COVID-19 pandemic [29].

One potential explanation is that participants in the second group significantly increased their intake of saturated fat, cholesterol and arachidonic acid (AA) [74]. AA, an omega-6 (n-6) polyunsaturated fatty acid (PUFA), is a precursor to a number of potent pro-inflammatory mediators, such as leukotrienes and prostaglandins [75, 76]. Pathologically elevated levels of these pro-inflammatory cytokines have been repeatedly associated with adverse effects on neurotransmitters [77] and elevated levels of inflammation were found in men with anxiety disorders [78]. The study by Beezhold et al. [74] suggests that even the higher dietary intake of fish-derived EPA and DHA, which are generally considered anti-inflammatory [79], failed to compensate for the harmful pro-inflammatory effects of arachidonic acid [73].

Of note, some authors also emphasized opposite findings and highlighted that fish oil supplementation is beneficial in the treatment of depression (when compared with placebo) [80]. The debate on potential therapeutic effects of omega-3 fatty acids in mental health disorders is ongoing, and randomized clinical trials have yielded inconclusive results [81]. Given the fact that the aforementioned supplements also bear the potential for unwanted side effects (particularly at higher dosage levels) [81, 82], it might be advisable to set the focus on a reduced intake of potentially pro-inflammatory mediators.

Over the past decades, dietary changes toward nutrition patterns high in processed and animal foods lead to striking increases in the (n-6) to (n-3) ratio (~ 15:1) [76], whereas plant-based diets were associated with more favorable fatty acid profiles [83]. Particularly, the vegan diet includes low proportions of AA [84], while simultaneously increasing PUFA intake (when expressed as a percent of total fat intake) [85]. These factors may be one reason why vegans report less stress and anxiety than omnivores [86], whereas pro-inflammatory Western diets were positively associated with depressive symptoms in both adults [87] and children [88].

In light of these findings, anti-inflammatory plant-based dietary patterns could be a potential tool to tackle the increasing public mental health burden associated with COVID-19. Although this nutritional approach has not yet been tested in COVID-19 cohorts, its neuroprotective effects (that is preserving the brain from inflammation and oxidative stress) warrant further consideration [89, 90]. Such a diet is also abundant in tryptophan, a key element for brain functioning, mental health prevention, and the precursor of serotonin [91, 92].

### Neurotransmitter Balance

Serotonin plays an essential role in the development of depression [59]. While serotonin itself cannot pass the blood–brain barrier [92], central nervous system (CNS) serotonin synthesis can be controlled by proper intake of a tryptophan-rich diet [93]. A 2015 study in 25 healthy young adults demonstrated that consuming more dietary tryptophan resulted in less depressive symptoms and decreased anxiety [94]. A tryptophan-rich diet is not only a potential protective factor against depression but also positively related to functioning in social cognition [95]. Particularly in the elderly, mild and moderate depression may be associated with a lower intake of tryptophan [96].

Several studies identified substantial alterations in tryptophan metabolism in COVID-19 patients [97, 98], and some experts suggested that COVID-19-related alteration in both tryptophan absorption and metabolism could be the underlying pathophysiology of long-COVID symptoms [99]. Thus, it appears conceivable to ensure an adequate tryptophan supply in affected individuals (particularly in those receiving interferons [100].

Tryptophan is transported into the brain via the leucine-preferring L1-system, where it competes with other large neutral amino acids (LNAA), such as valine, leucine, and isoleucine [101]. The tryptophan: LNAA ratio determines the flux of tryptophan into the brain and is of paramount importance for serotonin biosynthesis [92, 102].

Interestingly, different meals affect this ratio in different ways: a carbohydrate-rich meal increases this ratio while a protein-rich meal significantly decreases it [92, 103]. Carbohydrate-rich meals enhance insulin secretion and subsequently stimulate the clearance of branched amino acids (valine, leucine, isoleucine) from plasma [101]. Once these amino acids are absorbed by muscle cells, tryptophan availability for CNS uptake increases. Furthermore, vegan diets were generally found to be lower in valine and isoleucine (as compared to meat-rich diets) [104], a phenomenon that may beneficially affect tryptophan CNS uptake as well.

An often-recommended approach for ideal brain tryptophan levels is to focus on high-quality plant proteins along with generous amounts of (complex and unrefined) carbohydrates, as found in abundance in fruits, vegetable, and legumes [73].

## Sleep Quality

There is accumulating evidence that such a (tryptophan-rich) dietary pattern could also improve sleep quality and mitigate quarantine-related sleep problems [105, 106]. This appears of paramount importance as the COVID-19 pandemic is now a widely recognized risk factor for sleep disorders [107]. Lockdown periods were associated with later bedtime and waking time, an increase in daytime napping and a reduction in nighttime sleep [108, 109]. In light of the high number of individuals that experienced worse sleep during lockdown measures [110], cost-effective strategies to tackle this problem are urgently warranted.

Several studies (not directly related to COVID-19) suggest that a plant-based diet rich in fruits and vegetables can lead to improved sleep quality [111, 112]. The most recent example is the Helsinki Business study demonstrating an association between better sleep quality and vegetable consumption [113]. In contrast, a low-fiber and a high saturated fat intake were associated with lighter, less-restorative sleep [114].

A plant-based diet restricts (or avoids) foods that were associated with impaired sleep duration and quality, including meat [115] and high-fat products [116]. Yet, it is abundant in magnesium-rich foods [117], a nutrient associated with improvements in sleep quality, length of sleep time, and sleep onset latency as well as early morning awakening [118, 119].

Of note, an increased dietary magnesium intake could also benefit individuals with mental and physical stress [120, 121], two highly prevalent entities during the COVID-19 pandemic.

## Musculoskeletal Pain

Plant-based eating patterns could be generally useful to combat the skyrocketing prevalence of depression, anxiety, and sleep disorders associated with the COVID-19 pandemic. However, the benefits of a plant-based diet are not limited to mental health but may also improve a number of physical conditions associated with long-COVID.

A significant proportion of long-COVID patients report persistent muscle and joint pain [122, 123]. Nutrition was shown to play an important role in musculoskeletal well-being [124], as, for example, diets high in animal protein were linked to chronic pain [124]. Pain severity was also positively associated with a high fat and sugar intake [125] and a low fruit and fiber intake [126]. There is also evidence that a low intake of magnesium and folic acid contributes to chronic musculoskeletal pain [127], whereas a high magnesium intake confers protective effects [128].

A plant-based diet is abundant in these micronutrients and vitamins, while reducing (or completely restricting) animal products that are rich in pro-inflammatory metabolites and saturated fatty acids. After just 8 weeks, this particular dietary pattern improved musculoskeletal pain and functional limitations as well as quality of life in individuals suffering from chronic pain (as measured by the Short Form Health Survey and the Numeric Pain Rating Scale) in a 2018 study [129]. Moreover, a plant-based (vegan) diet improved functional status in severe osteoarthritis, rheumatoid arthritis [130] and chronic fibromyalgia symptoms [131].

Potential mechanisms of action include an increased intake of antioxidants [132] (with a subsequent neutralization of free radicals [129]), weight loss due to reduced caloric density [133, 134] (and a subsequent reduction in mechanical load), and finally, decreased exposure to pro-inflammatory precursors [129].

In patients with long-COVID, inflammatory pathways can remain perturbed up to 60 days after a SARS-CoV-2 infection [135•]. Adoption of a plant-based diet could be one accessible strategy to tackle this prolonged systemic inflammation often found in long-COVID [136]. The rationale behind this hypothesis is that plant-based diets have consistently been associated with favorable reductions in inflammatory biomarkers in numerous pre-pandemic studies [137–140].

Franco-de-Moraes et al. compared CRP levels in vegans, omnivores, and lacto-ovo-vegetarians [141]. CRP levels were significantly lower in vegans (0.5 mg/L (0.4–1.3)) compared to lacto-ovo-vegetarians (0.8 mg/L (0.4–1.7)) and omnivores (1.1 mg/L (0.6–2.2)). As shown by Shah and colleagues, an 8-week (low-fat) vegan intervention can significantly reduce high-sensitivity C-reactive protein by 32% [142•]. These findings are supported by a 2019 study demonstrating that adherence to a healthful plant-based diet was associated with lower hs-CRP concentrations [143].

Reviewing the potential role for immunologic aberrations and long-lasting inflammatory damage in post-COVID [1], it is biologically plausible that an anti-inflammatory plant-based diet could benefit affected individuals.

## Plant-Based Diets and the Immune Response

Plant-based (vegetarian) eating patterns beneficially affect biomarkers of inflammation and immune status [144]. Craddock et al. suggested that the high intake of some key nutrients and phytochemicals (e.g., resveratrol and quercetin) in groups following plant-based diets may favorably modulate their immune function [21]. While a plant-based diet cannot prevent an individual from developing COVID-19, it may lower the odds of developing moderate-to-severe COVID-19-like illness (as described in the chapter plant-based diets and COVID-19) [28••].

Plant-based dietary patterns are rich in antioxidants, phytosterols, and polyphenols, which positively affect several cell types implicated in immune function [145] and exhibit direct antiviral properties [146, 147]. Commonly consumed foods including vitamins A, D, and E and water-soluble constituents of mushrooms as well as polyphenols found in fruits and vegetables may improve natural killer cell functionality and activity [21, 148]. Studies dating back to 1989 found an increased natural killer cell activity of peripheral blood lymphocytes in vegetarian men (when compared with omnivores) [149]. A later study in vegans by Haddad et al., however, could not confirm these findings [150]. Yet, Haddad et al. also observed lower leukocyte counts in vegans [150], a finding that was confirmed in later studies [104, 151].

Craddock et al. hypothesized that this phenomenon in those following plant-based dietary patterns may be beneficial [21], as elevated leukocyte counts have been associated with increased risk of type-2-diabetes and metabolic syndrome [152, 153] — both also being significant risk factors for a severe COVID-19 disease [154]. Despite these findings, the effects of a plant-based on immune function are poorly understood and future studies are warranted to investigate whether such dietary patterns may truly protect from viral illnesses.

## Summary

Table 1 summarizes the main features of a plant-based diet and their potential benefits in patients with long-COVID.

**Table 1:** Features of a plant-based diet and their potential benefits in long-COVID-related symptoms

Features of plant-based diets	Potential benefits with regard to long-COVID-related symptoms
High in fiber [24–26]	A low fiber intake was associated with lighter, less restorative sleep [113]; increasing fiber consumption may be beneficial with regard to long-COVID-related sleep disorders
High in antioxidants and polyphenols (e.g., quercetin) [21, 52, 53]	Naturally-occurring dietary polyphenols exhibit antidepressant activity [42] and may be beneficial with regard to long-COVID-related mental health problems Antioxidants neutralize free radicals and may help to tackle prolonged systemic inflammation often found in long-COVID [136] Polyphenols positively affect several cell types implicated in immune function [145] and exhibit direct antiviral properties [146, 147]
Favorable fatty acid profile [83] (high in polyunsaturated fatty acids [85], low in saturated fat, cholesterol and arachidonic acid [84])	A high intake of (saturated) fat was associated with lighter, less restorative sleep [83]; reducing saturated fat intake could potentially improve long-COVID-related sleep disorders A reduced intake of pro-inflammatory precursor might help tackle prolonged systemic inflammation in long-COVID [136]
High in magnesium [117]	A higher dietary magnesium intake may improve sleep quality, length of sleep time, sleep onset latency [88] and may also alleviate mental and physical stress [89, 90]
Reduced caloric density [134], high nutrient density [155]	A plant-based diet may contribute to weight loss [134] and a subsequent reduction in mechanical load, an important feature with regard to long-COVID-related joint and muscle pain

## Strengths and Limitations

This narrative review has several strengths and limitations that warrant further discussion. To the best of our knowledge, it is the first narrative review that examined the potential role for plant-based eating patterns in long-COVID-related sequelae. The symptom-oriented approach covers some of the most commonly encountered complications found in patients with long-COVID. The present review also includes a broad spectrum of the literature and identified several (biologically) plausible mechanisms how a plant-based diet could potentially improve symptoms related to long-COVID.

At the same time, the exploratory nature of this review may be considered a major limitation. Clinical intervention studies investigating the effects of a plant-based diet in individuals with long-COVID are currently not available. Much of the authors’ hypothesis is undermined by (biologically) plausible information and mechanisms; however, the vast majority of the included studies in this review was published in the pre-pandemic era and was not done in patients with COVID-19. Whether results would have been the same (or better or worse) in this particular cohort remains subject to speculation. Some of the included studies in this narrative also share a common limitation of small sample size (e.g., [47, 74]), which may limit their validity. Finally, it is important to note that individuals consuming a plant-based diet often exhibit a healthier lifestyle in general [156], which includes higher rates of physical activity [157], higher education [157, 158], and lower smoking rates [159]. Such factors may be potential confounders that warrant consideration when interpreting the results of nutritional studies and could explain the better overall health of those consuming a plant-based diet.

## Conclusions and Future Direction

The SARS-CoV-2-pandemic has caused morbidity at an unprecedented global scale. Although additional research is warranted to understand the overall survivorship burden associated with long-COVID, its sequelae will most likely continue to increase in the foreseeable future. As such, healthcare systems and particularly outpatient infrastructures will face substantial challenges.

The pandemic has imposed a heavy economic burden on many countries and future public health policies might suffer from strict cost-control and rationing policies. Effective and economical strategies should therefore concomitantly tackle as many long-COVID symptoms as possible.

One such strategy could include the large-scale promotion of plant-based eating patterns, which have a substantial potential to improve both physical and mental conditions that are now frequently encountered in long-COVID. While few studies have (yet) investigated their effectiveness in this particular cohort, this review suggests that a plant-based eating pattern could potentially exert beneficial effects with regard to anxiety, depression, sleep disorders, musculoskeletal pain, and systemic inflammation — symptoms that are often reported by individuals suffering from long-COVID. Resource allocation should recognize this evidence and support clinical trials examining the role of plant-based nutrition and other dietary recommendations in the battle against long-COVID.

Future studies should include detailed macro- and micronutrient data and should particularly explore which plant-based eating patterns (e.g., lacto-ovo-vegetarian, vegan) confers the greatest benefits in the battle against long-COVID.

## Data Availability

Not applicable.
